# How (not) to argue about is/ought inferences in the cognitive sciences

**DOI:** 10.3389/fpsyg.2014.00503

**Published:** 2014-05-27

**Authors:** Katinka J. P. Quintelier, Lieuwe Zijlstra

**Affiliations:** ^1^Department of International Strategy and Marketing, Amsterdam Business School, University of AmsterdamAmsterdam, Netherlands; ^2^Department of Philosophy and Moral Sciences, Ghent UniversityGhent, Belgium

**Keywords:** is/ought gap, naturalistic fallacy, is/ought inferences, epistemic “oughts”, deontic “oughts”, defeasible reasoning, deontic reasoning

When scholars problematize is/ought inferences (IOI's), they sometimes refer to Hume's or Moore's fallacy (e.g., Schneider, 2000; Schroyens, 2009; Elqayam and Evans, 2011). Although inferring “ought” from “is” can be problematic, we argue that, in the context of contemporary IOI's in the cognitive sciences, invoking Hume or Moore might be misguided. This is because Hume's and Moore's arguments concern the validity and soundness of *deductive* inferences while in our view contemporary IOI's in the cognitive sciences are better interpreted as *defeasible* inferences.

In order to avoid misinterpretations, we first clarify key concepts in the debate in section Key Concepts. In section Mind the Gap, we revisit Hume's and Moore's arguments against inferring “ought” from “is,” and in section A Debate Shackled, we discuss contemporary IOI's in the cognitive sciences.

## Key concepts

Participants in the is/ought debate distinguish between descriptive statements and deontic statements. Descriptive statements describe or predict how the world is. Deontic statements prescribe or proscribe how we should act or reason.

While “is” statements are descriptive statements, “ought” statements can be descriptive as well as deontic. For instance, “the streets ought to be wet because it is raining” is a descriptive statement because it predicts that the streets will be wet. Conversely, “If you do not want to get wet, you ought to carry an umbrella,” is a deontic statement because it prescribes what you should do. In this comment, we only discuss “ought” statements as deontic statements. Accordingly, we will not discuss inferences from “is” to descriptive “oughts” (cf. Oaksford and Chater, 2009, 2011), but only inferences from “is” to deontic “oughts” (cf. Oaksford and Sellen, 2000; Stanovich and West, 2000).

We describe an is/ought inference as an attempt to evaluate (i.e., fine-tune, develop, arbitrate between) deontic statements on the basis of descriptive statements. The following is an example of an IOI:

(1) Premise: More intelligent people are more likely than less intelligent people to make a guess, instead of reason, when solving the Wason Selection Task.
  Conclusion: We ought to make a guess, instead of reason, when solving the Wason Selection Task.

This inference can be interpreted as a deductive argument. As such, the conclusion is true if the inference is valid and sound. A deductive inference is valid if the premises logically entail the conclusions, hence, if it is logically impossible for the premises to be true and the conclusion false. In this inference, it is possible that the premise is true while the conclusion is false. Thus, it is deductively invalid.

Soundness takes the actual truth of the premises (and conclusions) into account: An inference is sound if it is valid *and* all of its premises are true. The inference in this example is not sound because it is invalid. However, were it to be valid, it would still be unsound because the premise is false. More intelligent people are in fact more likely than less intelligent people to reason logically when solving the Wason Selection Task (Stanovich and West, 2000).

An inference can also be interpreted as a defeasible argument. Defeasible inferences have several features, two of which are relevant for our argument (cf. Pollock, 1987, 1992). First, the inference can be correct even if it is not deductively valid. Let us illustrate this features on the basis of the following inference (which is not an is/ought inference) (2):

(2) Premise: X looks red to me.
  Conclusion: X is red.

Clearly, the premise does not logically entail the conclusion. However, the inference is defeasibly correct because the premise supports the conclusion—most things that look red to me are, in fact, red.

A second feature of defeasible inferences is that, when the inference is correct, it can still be revised in the light of new information. For instance, if we learn that X is a daisy that is illuminated by red lights, which can make things appear red when they are not, we may suggest the following revised inference (3):

(3) Premise 1: X looks red to me.
  Premise 2: X is a daisy that is illuminated by red lights, which can make things appear red when they are not.
  Conclusion: X is not red.

While correct defeasible inferences can be revised in the light of new information, valid deductive inferences cannot: If the conclusion follows deductively from a (set of) premise(s), it will still follow deductively no matter how many premises we add. (This is termed the monotonicity of deductive logic.)

All this is relevant for is/ought debates. In section Mind the Gap, we argue that Hume's and Moore's arguments concern the validity and soundness of deductive inferences. In section A Debate Shackled, we explain why IOI's in the cognitive sciences are better interpreted—and evaluated—as defeasible inferences.

## Mind the gap

Cognitive scientists often fine-tune, develop or arbitrate between models of how people ought to reason on the basis of theories and data of how people do reason (for a discussion and critique, see Elqayam and Evans, 2011). Critics (e.g., Schneider, 2000; Schroyens, 2009; Elqayam and Evans, 2011) claim that some of these cognitive scientists commit Hume's or Moore's fallacy. However, in line with previous interpretations, we contend that Hume's and Moore's fallacies in the first place preclude deductive inferences that are, respectively, not valid and not sound (cf. Schurz, 1997; Pigden, 2010; Quintelier et al., 2011).

It is useful to introduce a caveat here. Hume and Moore formulated their arguments in the context of ethical “oughts.” However, in the cognitive sciences, their arguments are applied to epistemic “oughts.” This is acceptable for standard, logical, interpretations of Hume's fallacy, which seem to hold at least for deontic “oughts” in general (Pigden, 2010; P. 240). In contrast, it is unclear if Moore's fallacy applies to the same extent to non-ethical deontic “oughts.” For the sake of argument though, we assume that both fallacies also apply to epistemic “oughts.”

Let us now review Hume's fallacy. The standard interpretation of Hume's fallacy states that there are no deductively valid inferences whose premises contain no “oughts” and whose conclusions contain (non-trivial) “oughts” (Schurz, 1997; Pigden, 2010; p. 198–242). For example, the following inference is not deductively valid:

(3) Premise: It is the case that human beings apply Bayesian reasoning.
  Conclusion: It ought to be the case that human beings apply Bayesian reasoning.

This inference is not deductively valid because it is possible that the conclusions are false while the premises are true. In Hume's words, “ought, or ought not, expresses some new relation or affirmation,” which is different than the relation being expressed by “is,” or “is not” (1739–1740, Book III, Part I, section Key Concepts). When scholars infer “ought” related conclusions from premises that contain only “isses,” they commit Hume's fallacy.

However, Hume also argues that we can add a premise—hereafter termed a bridge principle - that connects “is” and “ought.” We can for example suggest the following bridge principle: “if more intelligent people apply reasoning X, we ought to apply reasoning X” (cf. Schneider, 2000, commenting on Stanovich and West, 2000). This principle can then be used as a premise:

(4) Premise 1: More intelligent people apply Bayesian reasoning.
  Premise 2: If more intelligent people apply Bayesian reasoning, we ought to apply Bayesian reasoning.
  Conclusion: We ought to apply Bayesian reasoning.

This inference is now deductively valid: if the premises are true, then the conclusion is also true. Hume's fallacy does not preclude the possibility of finding a plausible bridge principle.

In contrast, Moore's fallacy states that deductive IOI's with bridge principles might be valid, but they are never sound. The reason is that, according to Moore, bridge principles can never be true. Moore's argument is that we should find an analytically true bridge principle, one that spells out what descriptive concepts are in the meaning of the deontic concept (Moore, 1988, §1–15). However, pace Moore, this is impossible because deontic concepts are already simple terms; there is nothing in their meaning than the deontic concept itself. Therefore, there are no true bridge principles. Those who define a deontic concept in descriptive terms and then claim that this definition is analytically true, commit Moore's fallacy (id.).

To summarize, we hold that Hume's fallacy states that deductive IOI's are never valid without a bridge principle, while Moore's thesis states that deductive IOI's are never sound because there is no true bridge principle.

## A debate shackled

Invoking Hume's and Moore's fallacy to criticize IOI's in the cognitive sciences can be problematic: If, by making an is/ought inference, authors rarely mean to *deduce* deontic “oughts” from “isses,” then their IOI's should not be evaluated on the basis of their deductive validity or soundness. Indeed, we argue that it is more charitable to interpret contemporary IOI's in the cognitive sciences as defeasible inferences: Relevant authors (Oaksford and Sellen, 2000; Stanovich and West, 2000; Douven, 2011) point to descriptive reasons that suggest, rather than logically entail, deontic conclusions. Moreover, these authors aim to make correct inferences that are revisable in the light of new information. Let us take a look at these features of contemporary IOI's in the cognitive sciences.

Stanovich and West (2000) seem to endorse the following inference:

(5) Premise: Studies show that more intelligent people are more likely than less intelligent people to reason logically in task A.
  Conclusion: We ought to reason logically in task A.
  Oaksford and Sellen (2000) remark that the following also holds:
(6) Premise: Studies show that high schizotypal people are more likely than low schizotypal people to reason logically in task B.
  Conclusion: We ought not to reason logically in task B.

Clearly, these inferences are not deductively valid (cf. Schneider, 2000). However, these authors never claimed that their premise deductively entails a deontic conclusion. Instead, both Stanovich and West (2000; p. 645) and Oaksford and Sellen (2000; p. 691) speak of descriptive information that *suggests* a certain deontic conclusion. Moreover, these arguments are revisable in the light of new information: What if, for instance, both schizotypy and intelligence are positively correlated with logical reasoning in the same task A? In that case, we have to revise our conclusions that we ought to reason logically in task A. Thus, inferences 5 and 6 are better understood as defeasible inferences and ought to be evaluated accordingly.

Douven (2011) likewise suggests that, in certain cases, descriptive information can be used to inform us about deontic statements. He reasons as follows:

(7) Premise: Human beings update on conditionals by applying rule X.
  Conclusion: Human beings ought to update on conditionals by applying rule X.

Again, as a deductive inference, this would be invalid. However, Douven (2011) does not seem to have a deductive inference in mind. In his words, the premise again “suggests” the conclusion, and descriptive information leads to an “outline” of norms or, based on the premise, we can go “some way” in accepting the conclusion (253). This can be understood as a first approximation that can be revised. Moreover, there is no mentioning that descriptive premises logically entail a deontic conclusion.

These examples lead us to conclude that IOI's in the cognitive sciences are better interpreted as defeasible inferences than as deductive inferences. As a consequence, their deductive validity and soundness is not at stake. We therefore suggest that, instead of referring to Hume or Moore, critics of is/ought inferences apply evaluation criteria for defeasible inferences (see e.g., Nute, 1997). This conclusion supplements previous work on the is/ought problem. Schurz (in Pigden, 2010; p. 216), for instance, suggests that defeasible conditional norms might provide plausible bridge principles in ethical is/ought inferences. Other authors suggest that defeasible reasoning can solve problems and paradoxes occurring in monotonic deontic logic (e.g., Nute, 1997). However, previous work usually focused on ethical “oughts” rather than epistemic “oughts.” We therefore hope that this paper spurs research on defeasible reasoning with epistemic “oughts.”

### Conflict of interest statement

The authors declare that the research was conducted in the absence of any commercial or financial relationships that could be construed as a potential conflict of interest.
